# Altered connectivity patterns among resting state networks in patients with ischemic white matter lesions

**DOI:** 10.1007/s11682-017-9793-9

**Published:** 2017-11-14

**Authors:** Ju-Rong Ding, Xin Ding, Bo Hua, Xingzhong Xiong, Yuqiao Wen, Zhongxiang Ding, Qingsong Wang, Paul Thompson

**Affiliations:** 1 School of Automation and Information Engineering, Sichuan University of Science and Engineering, Zigong, China; 2 Imaging Genetics Center, Mark & Mary Stevens Institute for Neuroimaging and Informatics, University of Southern California, Marina del Rey, CA USA; 3 Department of Neurology, Chengdu Military General Hospital, Chengdu, China; 4 Department of Radiology, Zhejiang Provincial People’s Hospital, Hangzhou, China

**Keywords:** White matter lesions, Resting state networks, Functional connectivity, fMRI

## Abstract

White matter lesions (WMLs) have been associated with cognitive and motor decline. Resting state networks (RSNs) are spatially coherent patterns in the human brain and their interactions sustain our daily function. Therefore, investigating the altered intra- and inter-network connectivity among the RSNs may help to understand the association of WMLs with impaired cognitive and motor function. Here, we assessed alterations in functional connectivity patterns based on six well-defined RSNs—the default mode network (DMN), dorsal attention network (DAN), frontal-parietal control network (FPCN), auditory network (AN), sensory motor network (SMN) and visual network (VN)—in 15 patients with ischemic WMLs and 15 controls. In the patients, Spearman’s correlation analysis was further performed between these alterations and cognitive test scores, including Mini-Mental State Examination (MMSE) and Montreal Cognitive Assessment (MoCA) scores. Our results showed wide alterations of inter-network connectivity mainly involving the SMN, DMN, FPCN and DAN, and some alterations correlated with cognitive test scores in the patients. The reduced functional connectivities in the SMN-AN, SMN-VN, FPCN-AN, DAN-VN pairs may account for the cognitive and motor decline in patients with ischemic WMLs, while the increased functional connectivities in the DMN-AN, DMN-FPCN and DAN-FPCN pairs may reflect a functional network reorganization after damage to white matter. It is unexpected that altered intra-network connectivities were found within the AN and VN, which may explain the impairments in verbal fluency and information retrieval associated with WMLs. This study highlights the importance of functional connectivity in understanding how WMLs influence cognitive and behavior dysfunction.

## Introduction

Ischemic white matter lesions (WMLs) seen as white matter hyperintensities on T2-weighted magnetic resonance images (MRI) of the brain, are frequently observed in elderly individuals (Hachinski et al. 1987; Longstreth et al. 1996). WMLs are a neuroimaging biomarker of long-term cerebrovascular disease and are associated with heightened risk of dementia (Chutinet and Rost 2014).

In a number of cross-sectional and longitudinal studies, ischemic WMLs have been associated with cognitive and motor impairments (Longstreth et al. 1996; Silbert et al. 2008; Murray et al. 2010). However, it still remains unclear how ischemic WMLs alter an individual’s cognitive and motor function. Ischemic WMLs may result in disconnection of functionally related cortical regions, or they may impair the speed or integrity of signal transmission to cause cognitive impairments and eventually motor dyspraxias (Kim et al. 2008; Smith et al. 2011). Indeed, according to findings from basic and cognitive neurosciences, human cognition and behavior are subserved by multiple networks of interconnected neurons that enable parallel distributed signal processing (Mesulam 1990). Lesions in the white matter, therefore, may disrupt cortical perceptive and cognitive functions that are served by these networks (Kim et al. 2008). Moreover, in our recent works, we used resting state functional MRI (rs-fMRI) measures and found that patients with WMLs exhibited specific cortical dysfunction and disconnection related to cognitive and motor function (Ding et al. 2016, 2017). Rs-fMRI is a promising approach for characterizing functional connectivity in brain disorders and the good reproducibility of clinical findings has been reported in recent epilepsy studies (Zhu et al. 2015; Ji et al. 2017b). Thus, evaluating cortical functional connectivity based on rs-fMRI may improve our understanding of the association of WMLs with cognitive and motor decline.

In recent years, functional connectivity measures of rs-fMRI data have identified a set of spatially coherent patterns in the human brain, namely resting state networks (RSNs) (Damoiseaux et al. 2006; Fox and Raichle 2007; Smith et al. 2013). These RSNs are involved in multiple perceptive and cognitive functions, such as movement, vision, hearing, attention, executive control and episodic memory (Damoiseaux et al. 2006; Seeley et al. 2007; Ding et al. 2011). Functional interactions across all RSNs are thought to sustain our daily behavioral performances and emotional activities (Kelly et al. 2008; Liao et al. 2010b), and the disruption of RSNs associated with cognitive decline has been found in various disorders, such as multiple sclerosis (Rocca et al. 2014), major depression (Chen et al. 2016), post-traumatic stress disorder (Zhang et al. 2015) and Alzheimer’s disease (Brier et al. 2012). Therefore, investigation of RSNs may provide rich and sensitive information to understand mechanisms of disease (Castellanos et al. 2013; Smith et al. 2013).

In the present study, we aimed to explore the altered patterns in both intra-network and inter-network functional connectivities in patients with ischemic WMLs. Given the aforementioned studies, we hypothesized that these altered functional connectivity patterns would be related to cognitive and motor impairments in patients with ischemic WMLs. To test this theory, we investigated functional connectivity patterns for six well-defined RSNs including the default mode network (DMN), dorsal attention network (DAN), frontal-parietal control network (FPCN), auditory network (AN), sensory motor network (SMN) and visual network (VN). Since our recent works have found specific cortical dysfunction associated with motor, attention, memory and executive function in patients with WMLs (Ding et al. 2016, 2017), we further hypothesized that the interacting patterns across RSNs will be influenced in the patients, especially between DMN, DAN, FPCN and SMN. Finally, we tested how these altered functional connectivity patterns related to measures of cognitive performance.

## Materials and methods

### Ethics statement

The present study was approved by the Medical Ethics Committee of Chengdu Military General Hospital and were in accordance with the Declaration of Helsinki. Written informed consent was obtained from all participants before the study.

### Participants

**Fig. 1:**
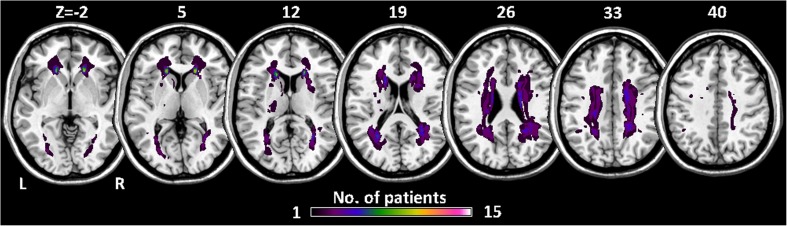
Spatial distribution of ischemic WMLs across all 15 patients. The colour code represents the number of patients in a given voxel. L, left; R, right

Some of the participants were the same as those assessed in our previous studies (Ding et al. 2016, 2017). The first group was composed of 17 patients (all right-handed, 7 males, age range: 49–72 years) who had been diagnosed clinically with ischemic WMLs. All participants underwent a comprehensive clinical examination by two experienced neurologists, including medical history, physical, and neurological assessments. They also finished laboratory examinations, such as a routine blood test, blood chemistry test, vitamin B12/folate measurement, human immunodeficiency virus infection screening, syphilis serology, and thyroid functioning tests. Patients with ischemic WMLs were determined based on their T2-weighted MRI images, defined as a ‘cap’ or a ‘band’ of 10 mm or more and a deep white matter lesion of 25 mm or more - according to a modification of the Fazekas ischemia criteria (Fazekas et al. 1993). A population-based probability map about the distribution of ischemic WMLs in the brain was produced using T2-weighted MRI images, and was presented in Fig. 1. Patients were excluded if they had: (1) psychiatric or neurological disorders that might cause cognitive impairment, such as stroke, schizophrenia, epilepsy, severe head trauma, encephalitis and brain tumors, (2) neurodegenerative diseases such as Parkinson’s disease, or (3) disorders that might impact their current cognitive state, including metabolic encephalopathy, human immunodeficiency virus infection, thyroid disease, syphilis, alcoholic encephalopathy and severe depression. The second group consisted of 16 controls (CN) (all right-handed, 8 males, age range: 54–71 years) with no WMLs on MRI. The control subjects had no neurological or psychiatric disorders. All participants in the two groups were evaluated with neuropsychological tests, including Mini-Mental State Examination (MMSE) (Folstein et al. 1975) and Montreal Cognitive Assessment (MoCA). Demographic and clinical characteristic of subjects are shown in Table 1.

**Table 1:** Demographic and clinical characteristics

Characteristics	Patients with WMLs (n = 15)	HCs (n = 15)	P-value
Age (years)	49–72 (61.7 ± 6.3)	54–71 (61.4 ± 5.4)	0.9017^a^
Gender (male/female)	7/8	8/7	> 0.9999^b^
Education (years)	6–15 (8.5 ± 2.9)	6–12 (8.1 ± 2.0)	0.8938^c^
Vascular risk factors			
Hypertension	7 (46.7%)	5 (33.3%)	0.7104^b^
Diabetes mellitus	2 (13.3%)	3 (20.0%)	> 0.9999^b^
Hyperlipidemia	6 (40.0%)	3 (20.0%)	0.4270^b^
Current smoker	1 (6.7%)	2 (13.3%)	> 0.9999^b^
MMSE	16–30 (23.7 ± 4.0)	27–29 (28.1 ± 1.0)	0.0009^d^
MoCA	10–24 (18.6 ± 4.1)	26–29 (27.1 ± 1.0)	< 0.0001^d^
Head motion	0.0857 ± 0.0425	0.0725 ± 0.0294	0.5338^c^

Data were expressed as the range from min-max (mean ± SD)

*Abbreviations: WMLs* white matter lesions, *HCs* healthy controls, *MMSE* mini-mental state examination, *MoCA* montreal cognitive assessment

^a^ *P*-value was obtained using the two-sample, two-tailed *t*-test

^b^ *P*-value was obtained using the two-tailed Fisher’s exact test

^c^ *P*-value was obtained using the two-tailed Mann-Whitney U test

^d^ *P*-value was obtained using the two-sample, two-tailed *t*-test with Welch’s correction

### Data acquisition

Imaging data were acquired on a 3.0-T Philips MR scanner (Philips Medical Systems, Best, Netherlands). During data acquisition, subjects were instructed to relax with their eyes closed, and move as little as possible. Foam padding and earplugs were used to reduce head motion and scanner noise. Functional data were collected as 35 transverse slices using an echo-planar-imaging sequence with the following acquisition parameters: repetition time = 2000 ms, echo time = 30 ms, flip angle = 90°, field of view = 192 × 192 mm^2^, matrix = 64 × 64, voxel size = 3 × 3 × 4 mm^3^, no inter-slice gap). A total of 230 volumes were acquired per subject. Additionally, a set of high-resolution T1-weighted anatomical images were also acquired in sagittal orientation using a 3D fast field echo sequence (repetition time = 2500 ms, echo time = 2.0 ms, flip angle = 30°, field of view = 192 × 256 mm^2^, matrix = 192 × 256, slice thickness = 1 mm, without inter-slice gap, voxel size = 1 × 1 × 1 mm^3^) for each subject.

### Data preprocessing

Data preprocessing was carried out using the toolbox for Data Processing & Analysis for Brain Imaging (DPABI V2.1, http://rfmri.org/dpabi) (Yan et al. 2016). After removing the first 10 volumes, the remaining 220 consecutive volumes were corrected for slice timing and realigned to the first volume. Then, several nuisance variables including a linear trend, the Friston 24-parameter model (Friston et al. 1996), white matter signal, cerebrospinal fluid signal and global mean signal were regressed from the realigned data (Yan et al. 2016). To better control for head motion effects, the time points with a threshold of frame-wise displacement (FD) > 0.2 mm as well as 2 forward and 1 back frames were also modeled as a scrubbing regressor in the multiple linear regression analysis (Power et al. 2012, 2013; Yan et al. 2016). Finally, the functional images were spatially normalized to the Montreal Neurological Institute (MNI) EPI template and temporally band-pass-filtered (0.01–0.1 Hz) to reduce effects of low-frequency drift and high-frequency noise (Cordes et al. 2001; Foerster et al. 2005).

### Quality control

Quality control was performed using DPABI toolbox. We visually inspected and rated the raw functional and normalized functional images. No participant was excluded due to bad scores on these images. Several previous studies indicate that even small amounts of head motion can affect estimation of functional connectivity in resting state fMRI studies (Power et al. 2012; Satterthwaite et al. 2012; Van Dijk et al. 2012). Therefore, we also excluded participants whose head motion (mean FD) was greater than 2*SD above the group mean motion (Yan et al. 2016). Consequently, two patients and one control subject were excluded from further analyses. Finally, 15 patients (7 males, age range: 49–72 years) and 15 controls (8 males, age range: 54–71 years) remained.

### Regions of interest definition

Regions of interest (ROIs) representing the DMN, DAN, FPCN, SMN and VN were defined based on prior studies of healthy subjects (Vincent et al. 2008; Gao and Lin 2012). Since our previous study has found that WMLs were related to abnormal functional connectivity density in temporal cortex (Ding et al. 2016), we also included the AN in subsequent network connectivity analyses. Overall, 36 spherical ROIs (with 6 mm radius) were obtained using the MarsBaR toolbox (MarsBaR 0.44, http://marsbar.sourceforge.net/) (for details see Table 2). The individual mean time series were then extracted for each ROI. Finally, Pearson’s correlation coefficients were calculated between each pair of ROIs for each subject, which resulted in a square 36 × 36 correlation matrix.

**Table 2:** Definition of regions of interest within six RSNs

RSN	Regions of interest	MNI coordinates
DMN	Left hippocampal formation (lHF)	− 21,− 15,− 14
	Right hippocampal formation (rHF)	24,− 19,− 21
	Ventromedial prefrontal cortex (vmPFC)	0,51,− 7
	Posterior cingulate cortex (PCC)	1,− 55,17
	Left posterior inferior parietal lobule (lpIPL)	− 47,− 71,29
	Right posterior inferior parietal lobule (rpIPL)	50,− 64,27
DAN	Left middle temporal area (lMT)	− 45,− 69,− 2
	Right middle temporal area (rMT)	50,− 69,− 3
	Left intraparietal sulcus (lIPS)	− 27,− 52,57
	Right intraparietal sulcus (lIPS)	24,− 56,55
	Left frontal eye field (lFEF)	− 25,− 8,50
	Right frontal eye field (rFEF)	27,− 8,50
FPCN	Left anterior prefrontal cortex (laPFC)	− 36,57,9
	Right anterior prefrontal cortex (raPFC)	34,52,10
	Anterior cingulate cortex (ACC)	3,31,27
	Left anterior inferior parietal lobule (laIPL)	− 52,− 49,47
	Right anterior inferior parietal lobule (raIPL)	52,− 46,46
	Left dorsolateral prefrontal cortex (ldlPFC)	− 50,20,34
	Right dorsolateral prefrontal cortex (rdlPFC)	46,14,43
	Left insula (lINS)	− 31,21,− 1
	Right insula (rINS)	31,22,− 2
AN	Left superior temporal gyrus (lSTG)	− 57,− 24,13
	Right superior temporal gyrus (rSTG)	60,− 24,13
	Left Heschl’s gyrus (lHes)	− 45,− 15,9
	Right Heschl’s gyrus (rHes)	45,− 18,9
SMN	Left precentral gyrus (lPreC)	− 41,− 4,54
	Right precentral gyrus (rPreC)	42,− 13,53
	Left postcentral gyrus (lPoC)	− 45,− 26,54
	Right postcentral gyrus (rPoC)	49,− 27,53
	Supplementary motor area (SMA)	6,− 5,54
VN	Left calcarine fissure (lCal)	− 8,− 72,4
	Right calcarine fissure (rCal)	16,− 67,5
	Left cuneus (lCS)	− 5,− 96,12
	Right cuneus (rCS)	18,− 96,12
	Left lateral occipital (lLO)	− 23,− 89,12
	Right lateral occipital (rLO)	37,− 85,13

*Abbreviations: RSNs* resting state networks, *MNI* montreal neurological institute, *DMN* default mode network, *DAN* dorsal attention network, *FPCN* frontal-parietal control network, *AN* auditory network, *SMN* sensorimotor network, *VN* visual network

### Functional connectivity analyses

Prior to investigating functional connectivity patterns in patients with ischemic WMLs, the correlation coefficients were converted to $\eta$ values using an exponential function related to the connectivity distance between the two connected ROIs (Lopez and Sanjuan 2002). The conversion formula was ${\eta _{{\text{ij}}}}={e^{ - \xi {d_{ij}}}}$, where $\xi$ is a positive constant measuring how the strength of the relationship decreases with the distance between two ROIs, and is here set to 2 according to the study from Lopez and Sanjuan (2002); ${d_{ij}}={{\left( {1 - {r_{ij}}} \right)} \mathord{\left/ {\vphantom {{\left( {1 - {r_{ij}}} \right)} {\left( {1+{r_{ij}}} \right)}}} \right. \kern-0pt} {\left( {1+{r_{ij}}} \right)}}$ is a hyperbolic correlation measure representing the distance between ROI $i$ and ROI $j$, ${r_{ij}}$ is Pearson’s correlation coefficient (Lopez and Sanjuan 2002). This processing step was to ensure that all correlations were positive, which could be better to deal with the influence of negative correlations when computing intra- and inter-network connectivity.

The human brain is a complex network. Cognitive and behavior abnormalities are related not only to specific cortical dysfunction but also to altered interacting patterns between subnetworks – RSNs (van den Heuvel and Hulshoff Pol 2010). Therefore, we analyzed functional connectivity patterns at the following three levels in this study.

1. Nodal integration level

For each node (here an ROI) $i$, the integration was computed as ${\Gamma _i}=\sum\nolimits_{{j=1}}^{n} {{\eta _{ij}}}$(Lopez and Sanjuan 2002; Jiang et al. 2004), where $n$ refers to the number of nodes and is 36 in this study. The nodal integration describes the capacity for integrating information from all other nodes of a network. It may be possible to investigate alterations in the total functional connectivity degree in different brain activity states (Jiang et al. 2004).

(2) Network level

As prior studies described (Brier et al. 2012; Chen et al. 2016), the intra-network composite score was computed as the mean transformed correlation coefficients of all ROI pairs in the same RSN, that is, $c_{k}^{X}={\left\langle {{\eta _{ijk}}} \right\rangle _{i,j \in X}}$, where $k$ represents a subject, $i$ and $j$ indicate an ROI pair within a RSN $X$ and 〈〉 denotes the mean across ROI pairs. Similarly, the inter-network composite score was defined as the mean transformed correlation coefficients of all ROI pairs belonging to different RSNs, that is, $c_{k}^{{X,Y}}={\left\langle {{\eta _{ijk}}} \right\rangle _{i \in X,j \in Y}}$, where $X$ and $Y$ represent different RSNs.

(3) Connectivity level

To identify specific functional connectivity with significant between-group differences, we used a network-based statistic (NBS) approach proposed by Zalesky et al. (Zalesky et al. 2010) This method provides a solution to the statistical problem of a massive number of multiple comparisons. A detailed description of this method can be found in the study of Zalesky and colleagues (Zalesky et al. 2010). Briefly, an F-test, followed by one-sided *t*-tests (after controlling for the effects of age and sex) was performed on each of the $36 \times (36 - 1)/2=630$ pairs of ROIs. Pairs of ROIs with a test statistic exceeding a threshold $F$ were admitted to the set of suprathreshold links where any connected components and their size (the number of links in these components) were determined. To evaluate the significance for each component, a total of 5,000 permutations were generated to estimate the null distribution of the maximal component size. Finally, a corrected *p* value for a connected component of size *M* found in the original data was computed as the proportion of 5,000 permutations for which the largest connected component was larger than *M*. The significant group differences in connectivity were visualized using BrainNet Viewer (Xia et al. 2013).

### Statistical analysis

For nodal integration and network level, two-sample two-tailed *t*-tests were used to assess group differences after controlling for the effects of age and sex. The statistical significance for these group comparisons was determined using permutation tests. In the present study, permutations were performed 5,000 times to test whether the group differences were significant. The significant threshold was set to $\alpha =0.05$ with the false discovery rate (FDR) correction for multiple comparisons. Meanwhile, a lenient significant level $p<0.05$ (uncorrected) was also performed for an exploratory investigation. At connectivity level, statistically significant group differences were identified at a threshold $F$ ($F=5.3$ in the present study) with correction for multiple comparisons at $p<0.05$ using the NBS method as described above.

To relate cognitive performance to altered connectivity patterns at the three levels, Spearman correlation analysis was further performed to test for associations between these alterations and cognitive test scores (MMSE scores and MoCA scores) in patients with ischemic WMLs. As this analysis was exploratory, a significance threshold of $p<0.05$ (uncorrected) was used.

## Results

### Demographic and clinical characteristics

As seen in Table 1, there were no significant differences between the two groups in terms of age, sex, educational level, vascular risk factors, and head motion. With respect to cognitive performance, MMSE and MoCA scores were significantly lower in the patient group compared to the CN group, suggesting an obvious cognitive impairment in the patients with ischemic WMLs.

### Alterations on nodal integration level

**Fig. 2:**
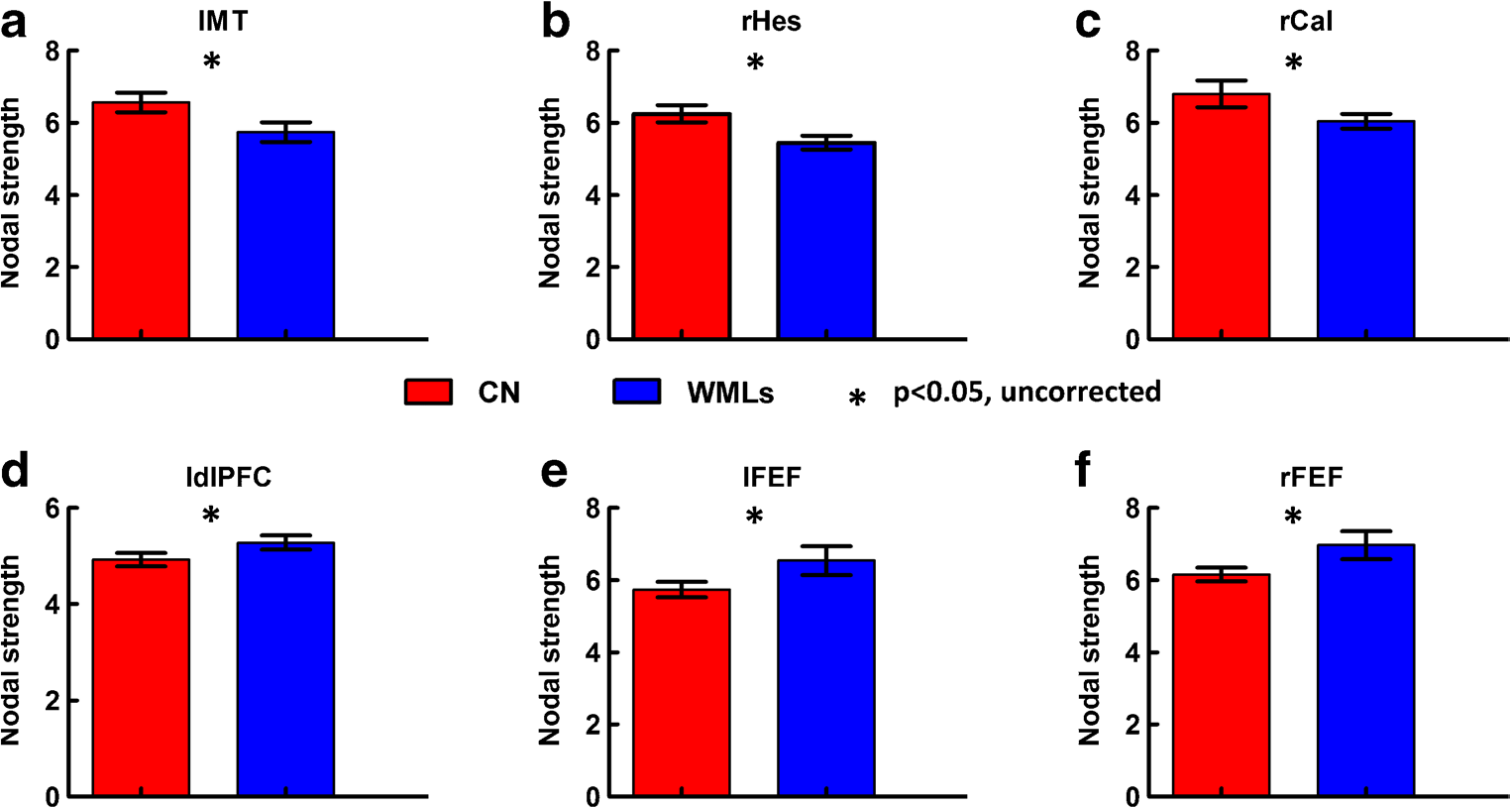
Statistical group differences of nodal strength at the nodal integration level. The asterisk indicates $p<0.05$ (uncorrected). WMLs, white matter lesions; CN, controls; MT, middle temporal area; Hes, Heschl’s gyrus; Cal, calcarine fissure; dlPFC, dorsolateral prefrontal cortex; FEF, frontal eye field, l, left; r, right

Group differences of nodal integration only survived under the lenient significant level of ${p_{uncorrected}}<0.05$ (Fig. 2). The patients exhibited obviously reduced nodal strength in the lMT, rHes, rCal; and increased nodal strength in the ldlPFC, lFEF and rFEF compared to the controls. No significant correlations were found between these regions and cognitive test scores.

### Alterations on network level

**Fig. 3:**
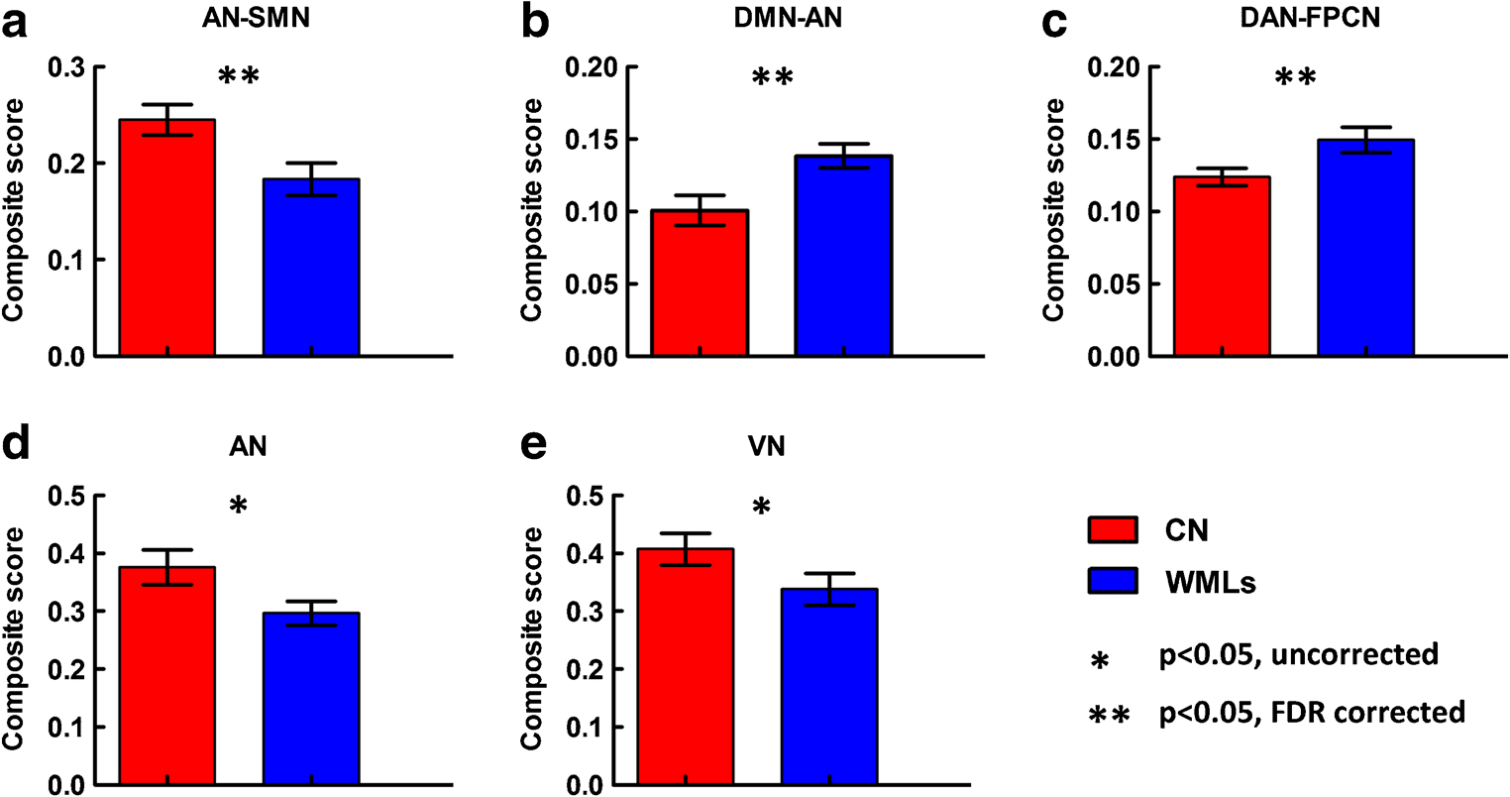
Statistical group differences in intra- and inter-network connectivity at the network level. **a**–**c** Inter-network connectivity of the AN-SMN, DMN-AN and DAN-FPCN pair, respectively. **d** and **e** Intra-network connectivity of the AN and VN, respectively. The double-asterisk indicates $p<0.05$ (FDR corrected). The asterisk indicates$p<0.05$ (uncorrected). WMLs, white matter lesions; CN, controls; AN, auditory network; VN, visual network; SMN, sensory motor network; DMN, default mode network; DAN, dorsal attention network; FPCN, frontal-parietal control network

Compared to the controls, the patients showed significantly lower inter-network composite score in the AN-SMN pair, and higher composite score in the DMN-AN pair and DAN-FPCN pair, respectively ($p<0.05$, FDR corrected) (Fig. 3a–c). In addition, using a lenient significant level of ${p_{uncorrected}}<0.05$, the composite score was further found to be lower within the AN and VN in the patient group (Fig. 3d and e). No significant correlations with cognitive test scores were detected.

### Alterations on connectivity level

**Fig. 4:**
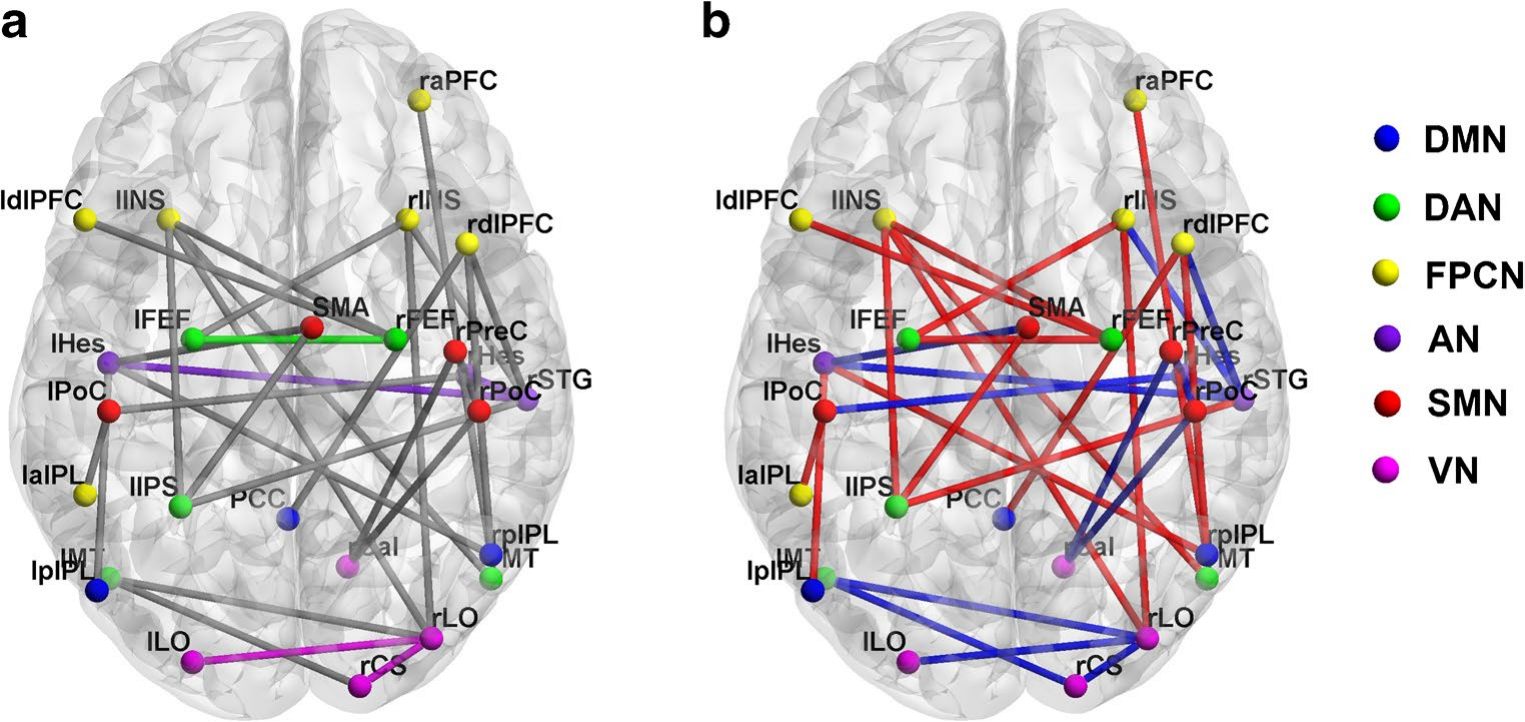
Distribution of altered functional connectivities at the network level in the patient group, identified with the NBS approach (${p_{NBS}}<0.05$). **a** Gray lines indicate that the connected nodes belong to different RSNs, while the colored lines indicate that the nodes belong to the same RSN. **b** Red and blue lines denote significantly higher and lower functional connectivity in the patients, respectively. AN, auditory network; VN, visual network; SMN, sensory motor network; DMN, default mode network; DAN, dorsal attention network; FPCN, frontal-parietal control network; SMA, supplementary motor area; PreC, precentral gyrus; PoC, postcentral gyrus; STG, superior temporal gyrus; Hes, Heschl’s gyrus; Cal, calcarine fissure; LO, lateral occipital; CS, cuneus; PCC, posterior cingulate cortex; pIPL, posterior inferior parietal lobule; INS, insula; aPFC, anterior prefrontal cortex; dlPFC, dorsolateral prefrontal cortex; aIPL, anterior inferior parietal lobule; IPS, intraparietal sulcus; MT, middle temporal area; FEF, frontal eye field; left; r, right

As seen in Fig. 4, thirty intra- and inter-network functional connectivities were altered in the patient group, based on using the NBS approach with a $p<0.05$ corrected for multiple comparisons. Consistent with our hypothesis, these altered connectivities were mainly among the SMN, DMN, FPCN and DAN. Specifically, the lower functional connectivities mainly involved the SMN-AN, SMN-VN, FPCN-AN and DAN-VN pairs. Apparently increased functional connectivities were mainly distributed in the DMN-AN, DMN-FPCN and DAN-FPCN pairs. It is interesting that the altered intra-network connectivities were located within the AN and VN.

**Fig. 5:**
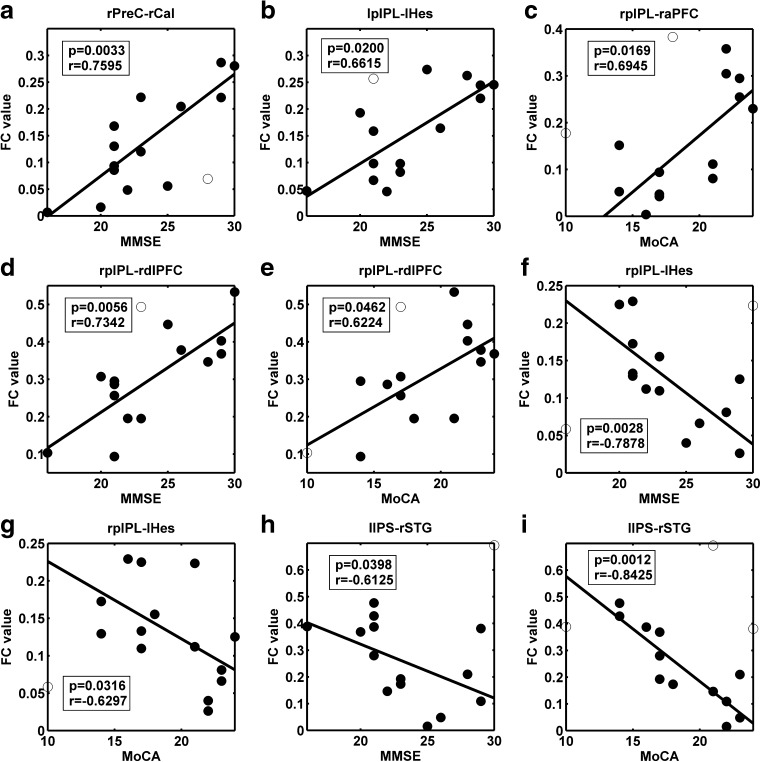
Correlations between altered functional connectivities and MMSE and MoCA scores in the patient group (${p_{uncorrected}}<0.05$). Spearman correlations were computed over the data after removing outliers marked by circles. MMSE, Mini-Mental State Examination; MoCA, Montreal Cognitive Assessment; FC, functional connectivity; PreC, precentral gyrus; Cal, calcarine fissure; Hes, Heschl’s gyrus; pIPL, posterior inferior parietal lobule; aPFC, anterior prefrontal cortex; dlPFC, dorsolateral prefrontal cortex; IPS, intraparietal sulcus; STG, superior temporal gyrus; l, left; r, right

We further performed Spearman correlations to investigate the association between these altered functional connectivities and cognitive test scores (MMSE and MoCA scores) in the patient group, after removing potential outliers (Schwarzkopf et al. 2012). The reduced functional connectivity between the rPreC-rCal pair and increased functional connectivity between the lpIPL-lHes pair were positively correlated with MMSE scores, respectively (Fig. 5a and b). The increased functional connectivity between the rpIPL-raPFC pair was positively correlated with MoCA scores (Fig. 5c). For both MMSE and MoCA scores, the increased functional connectivity between the rpIPL-rdlPFC pair showed a positive correlation, while the rpIPL-lHes and lIPS-rSTG pairs exhibited a negative correlation, respectively (Fig. 5d–i).

## Discussion

In this study, we sought to assess changes in intra-network and inter-network functional connectivity patterns in patients with ischemic WMLs. At the nodal integration level, the regions with altered nodal strength in the patient group were mainly located in the AN, VN, DAN and FPCN. Further analyses at the network and connectivity levels revealed lower functional connectivities in the patients, mainly in the AN, VN, AN-SMN, SMN-VN, FPCN-AN and DAN-VN pairs, and increased functional connectivities in the DMN-AN, DMN-FPCN and DAN-FPCN pairs. In addition, some of the altered functional connectivities were correlated with cognitive test scores. These findings may aid to reveal the relationship of ischemic WMLs with individuals’ cognitive and motor function.

### Altered functional connectivity within RSNs

The RSNs reflect specific perceptual and cognitive functions of the human brain, and exploring intra-network connectivity of RSNs can help us to understand behavioral dysfunction and cognitive impairments related to disease (Liao et al. 2010a; Castellanos et al. 2013; Smith et al. 2013). In the present study, reduced intra-network connectivity was found within the AN and VN in patients with ischemic WMLs. The AN and VN are primary perceptual networks, and they participate in receiving external signals, selecting relevant information and conveying them to the higher cognitive networks to induce appropriate responses (Mesulam 1998). In our connectivity analyses, we found that reduced functional connectivities within the AN were mainly among the rSTG and bilateral Hes, supporting and extending our prior findings of reduced functional connectivity density in the temporal cortex (Ding et al. 2016). The STG and Hes are involved in speech and language-related processing (Bigler et al. 2007; Warrier et al. 2009), and the rSTG has been noted as playing a key role in social cognition, as well as in processing and integrating various information to give reasonable response to external stimuli (Allison et al. 2000; Zilbovicius et al. 2006). Besides, the reduced functional connectivities within the VN were found among the bilateral LO and rCS. The CS participates in basic visual information processing, and the LO is implicated in object recognition and visual perception (Grill-Spector and Malach 2004). Therefore, the reduced functional connectivity within the AN and VN probably affect the perception and integration of language-related information, which may account for the impairments in verbal fluency and information retrieval in patients with WMLs (Bolandzadeh et al. 2012).

### Altered functional connectivity between RSNs

Individuals’ daily cognitive function and behavior depend on both focal processing of specific brain regions and global integration of neuronal activity (Mesulam 1990; Tononi et al. 1998). Intra-network connectivity measures within the RSNs allow us to assess functional specialization in the brain, whereas inter-network measures between the RSNs can help us assess global integration.

In this study, inter-network connectivity was reduced in the AN-SMN pair in patients with ischemic WMLs. At the connectivity level, the reduced functional connectivities were also found between the SMN and VN. The significantly reduced functional connectivities were mainly involved in the SMA-lHes, rPoC-rHes, rPreC-rHes, rPoC-rCal and rPreC-rCal. The SMA plays an important role in postural stabilization, voluntary movements, bimanual coordination, retrieving correct actions based on memory (Shima and Tanji 1998; Graziano and Aflalo 2007). The PreC is the primary motor cortex, which is involved in executing voluntary movements, while the PoC is the primary somatosensory cortex; lesions to this area may cause somatosensory disturbances such as tactile discrimination and postural sensitivity (Bigbee 2011). Generally, the AN and VN are involved in receiving, selecting, and conveying external information to induce responses, and the SMN is responsible for executing responses for external stimuli and internally generated movements. To some extent, the reduced inter-network connectivity among the SMN, AN and VN may be attributed to the disrupted intra-network functional connectivity of the AN and VN. Our findings may be possible explanations for the gait and balance impairments associated with WMLs (Starr et al. 2003). Moreover, the reduced functional connectivity of the rPreC-rCal was positively correlated with MMSE scores, supporting that cognitive capacity can affect individuals’ behavior performances (Starr et al. 2003).

In our connectivity analyses, the patients also showed reduced functional connectivities between the FPCN (rdlPFC and rINS) and AN (rSTG), as well as between the DAN (lMT) and VN (rLO and rCS). Prior studies found that the dlPFC is involved in executive function and working memory (Pochon et al. 2001), and the INS is related to emotional regulation, self-awareness and language function (Craig 2002; Ibanez et al. 2010). The MT is an important region of the DAN and associated with visuospatial attention (Vincent et al. 2008). Thus, these reduced functional connectivities may provide an explanation for the impairments in verbal fluency, attention, memory and executive function associated with WMLs (Kramer et al. 2002; Nordahl et al. 2006; Xiong and Mok 2011; Bolandzadeh et al. 2012).

Higher inter-network connectivity was found in the DAN-FPCN pair, as well as between the DMN and FPCN at the connectivity level in the patient group. The DAN is associated with externally directed cognition, such as eye movements, allocating attentional resources, generating motor plans (Corbetta and Shulman 2002), while the DMN is involved in internally directed cognition, including mind wandering, self-reference, episodic memory and environmental monitoring (Raichle et al. 2001; Buckner et al. 2008). The FPCN is considered to participate in executive function, cognitive control and top-down modulation of attention and memory retrieval (Koechlin et al. 1999; Vincent et al. 2008). The FPCN may have an intermediate role interacting with the DMN and DAN, and it may couple its activity with either of them in support of goal-directed cognition (Vincent et al. 2008; Gao and Lin 2012). White matter damage may diminish the efficiency of neural transmission and then reduce cortical functional connectivity (Reed et al. 2004). Even so, in some cases, cortical plasticity may enable the brain to reorganize by forming new neural or strengthened functional connections (Nudo et al. 1996; Buonomano and Merzenich 1998; Dancause et al. 2005). Therefore, we speculate that the increased functional connectivity in the DAN-FPCN and DMN-FPCN pairs may reflect a functional network reorganization to compensate for the cognitive decline in patients with ischemic WMLs. Furthermore, the increased functional connectivities between the DMN and FPCN (the rpIPL-rdlPFC and rpIPL-raPFC) exhibited positive correlations with the MMSE and MoCA scores, which may support this speculation. The higher strength of functional connectivity corresponds to a greater compensation, which induces a better cognitive function.

In addition, the increased inter-network connectivity was also found in the DMN-AN pair in patients with ischemic WMLs. Using Granger causality analyses, our prior study also observed the interaction between the DMN and AN in healthy subjects (Liao et al. 2010b). Tian et al. (Tian et al. 2007) reported stable negative correlations between the DMN and AN during either task-backgrounds (periods not performing a task in a task-related study), or in the resting state. However, this interaction could be influenced by noise from the scanner (Tian et al. 2007; Liao et al. 2010b). Here, we further found that the increased functional connectivity between the lpIPL and lHes was positively correlated with MMSE scores, but functional connectivity between the rpIPL and lHes showed a negative correlation with the MMSE and MoCA scores. These findings indicate that the interaction between the DMN and AN in patients with ischemic WMLs should be carefully understood, and may not just be due to scanner noise. Further studies are needed to investigate this interaction.

There are some future considerations and methodological limitations in this study. The recent rs-fMRI studies have demonstrated that the blood oxygen level dependent (BOLD) signal fluctuations in white matter have biological sources similar to those in grey matter (Ji et al. 2017a; Peer et al. 2017). Future studies, therefore, may directly use the resting state BOLD signal of white matter to investigate functional connectivity in patients with ischemic WMLs, which can provide more direct evidence of how information is affected by lesions in white matter. In addition, transcranial magnetic stimulation—a non-invasive brain stimulation technique—has been found to be of potential value for improving clinical symptoms in neurological disorders by affecting functional connectivity of target regions with other brain areas (Edwards et al. 2008; Fox et al. 2012; Le et al. 2013; Ji et al. 2016). The regions with abnormal functional connectivity found in this study, especially these superficial regions (e.g., SMA, PoC, PreC, dlPFC), may be tested as potential targets for non-invasive treatment of ischemic patients in future studies. Our study has some methodological limitations. First, the sample size is relatively small. A larger sample size is needed to increase statistical power for future studies. Second, the connectivity analyses were conducted on six well-defined RSNs. The human brain is a dynamic, complex system organized by multiple distinct and interacting subnetworks. Future research should include other subnetworks to explore more wide connectivity alterations associated with WMLs. Third, the Pearson’s correlation coefficients were converted into a new index using an exponential function (Lopez and Sanjuan 2002). This conversion ensured that all the correlations were positive and avoided the offsetting of positive and negative correlations when computing the nodal integration and network composite scores (Chen et al. 2016). However, negative correlations have been found exist between different RSNs, such as between DMN and DAN, with or without global signal regression, and might have a biological basis (Fox et al. 2009; Chai et al. 2012). Therefore, the present findings should be interpreted accordingly.

## Conclusions

In summary, we revealed widely altered patterns of intra- and inter-network connectivity among the RSNs in patients with ischemic WMLs. The reduced functional connectivities were located within the AN and VN, as well as among the SMN, AN, VN, FPCN and DAN. These alterations may accompany the cognitive and motor decline in patients with ischemic WMLs. The increased functional connectivities among the FPCN, DMN and DAN may reflect the reorganization of functional networks to compensate for the cognitive impairments associated with WMLs. These connectivity pattern alterations and their associations with cognitive performance may play a vital role in understanding the association of WMLs with cognitive and motor decline.
